# Parametric Estimation of the Mean Number of Events in the Presence of Competing Risks

**DOI:** 10.1002/bimj.70038

**Published:** 2025-02-18

**Authors:** Joshua P. Entrop, Lasse H. Jakobsen, Michael J. Crowther, Mark Clements, Sandra Eloranta, Caroline E. Dietrich

**Affiliations:** ^1^ Clinical Epidemiology Division Department of Medicine Solna, Karolinska Institutet Stockholm Sweden; ^2^ Department of Hematology Clinical Cancer Research Center Aalborg University Hospital Aalborg Denmark; ^3^ Department of Mathematical Science Aalborg University Aalborg Denmark; ^4^ Red Door Analytics AB Stockholm Sweden; ^5^ Department of Medical Epidemiology and Biostatistics Karolinska Institutet Stockholm Sweden

**Keywords:** competing events, flexible parametric survival models, recurrent events, survival analysis

## Abstract

Recurrent events, for example, hospitalizations or drug prescriptions, are common in time‐to‐event research. One useful summary measure of the recurrent event process is the mean number of events. Methods for estimating the mean number of events exist and are readily implemented for situations in which the recurrent event is the only possible outcome. However, estimation gets more challenging in the competing risk setting, in which methods are so far limited to nonparametric approaches. To this end, we propose a postestimation command for estimating the mean number of events in the presence of competing risks by jointly modeling the intensity function of the recurrent event and the survival function for the competing events. The proposed method is implemented in the R‐package JointFPM which is available on CRAN. Simulations demonstrate low bias and good coverage in scenarios where the intensity of the recurrent event does not depend on the number of previous events. We illustrate our method using data on readmissions after colorectal cancer surgery included in the frailtypack package for R. Estimates of the mean number of events can be used to augment time‐to‐event analyses when both recurrent and competing events exist. The proposed parametric approach offers estimation of a smooth function across time as well as easy estimation of different contrasts which is not available using a nonparametric approach.

## 1 Introduction

Analyses of time‐to‐event data typically focus on the time until the first occurrence of an event, even when the event can occur multiple times. Including not only the first but also subsequent events, that is, performing a recurrent events analysis, might reveal more insight than the standard approach as the number of events can be useful as, for example, a marker of event intensity or severity. Examples where this is relevant include the study of hospitalization due to chronic obstructive pulmonary disease (COPD) among individuals with respiratory impairment (Fragoso et al. 2012). Given that hospitalizations due to COPD are typically reoccurring, not only time to first hospitalization is important in relation to respiratory impairment, but also the mean number of hospitalizations an individual might encounter. Another example where conducting a recurrent event analysis might provide a more complete picture is in the comparison of (repeated) COVID infections in clinical trials by different vaccines (Polack et al. 2020). Other commonly occurring examples include cardiovascular disease, childbirths, and cancer relapses.

The most common approach to incorporate recurrent events in a time‐to‐event analysis is by estimating transition‐specific models and associated transition probabilities. This is typically done using either multistate models, longitudinal models, or extensions of the Andersen–Gill Cox model (Cook and Lawless 2010). Although estimates from these models can provide a detailed picture of the event process, modeling, and interpretation becomes challenging when the occurrence of events is high. Estimates of the marginal mean number of events provide a useful absolute summary measure of the event process with an intuitive interpretation, which does not require the specification of the event process for each event occurrence.

Recurrent events often occur in situations where some competing event can prevent the (next) recurrent event from occurring. For example, death due to any cause is a competing event in the above‐mentioned example on COPD hospitalization, that *prevents* individuals from being hospitalized with COPD (Fragoso et al. 2012). Note that a competing event does not always have to be a terminal event. Geskus (2016), for example, describes a study on the risk of hospital‐acquired infections in which hospital discharge is a competing yet not terminal event, as discharge from the hospital is not preventing individuals from having infections later. However, hospital discharge prevents individuals from getting hospital‐acquired infections in the hospital under study, assuming that the observational units are hospital admissions. In these situations in which both a recurrent and a competing event are present, cause‐specific cumulative incidence functions (CIFs) of the first event are commonly reported. Reporting CIFs in these situations disregards the recurrent nature of the event of interest and also changes the questions of interest from a question about hospitalizations to one about first hospitalizations, when both questions might be of interest.

Methods for jointly modeling the recurrent and competing event are widespread and highly developed, albeit mainly framed in a frailty or multistate model setting (Chauvet and Rondeau 2023; Putter, Fiocco, and Geskus 2007). As pointed out by Cook and Lawless, there are four types of models for the analysis of recurrent events in the competing risk setting (Cook and Lawless 2010):

1. *Intensity‐based* approaches in which recurrent events and competing risks are modeled using a joint counting process model for both the counting process of the recurrent and the competing events;
2. *Random effect models* for jointly modeling the recurrent and competing event (Chauvet and Rondeau 2023);
3. *Partially conditional models* which are a special case of multistate models in which each new event occurrence is defined as a new state;
4. *Marginal models* which model the average number of events at different time points and are marginal over the episode‐specific intensity process.

Estimation of models 1–3 requires that all transition rates between states are correctly specified, in contrast to *marginal models* which neither require specification of transition functions nor a maximum number of recurrent events.

While fully parametric estimation methods are readily available for joint frailty models and multistate models, such methods are sparse for marginal models. Ghosh and Lin (2000) discussed a nonparametric estimator of the mean number of events in the presence of competing risks, building on work by Cook and Lawless (1997). Cook and Lawless's estimator is an extension of the Aalen–Johansen (AJ) estimator of the cause‐specific CIF to the recurrent event setting. They showed that the AJ estimator can be used to estimate the mean number of events in the presence of competing risks by substituting the cause‐specific hazard function with a cause‐specific intensity function for the recurrent event (Ghosh and Lin 2000). Ghosh and Lin later introduced a semiparametric estimator of the mean number of events (Ghosh and Lin 2002). Fully parametric models for estimating the mean number of events are yet to be introduced for this setting, offering the possibility to estimate smooth functions of the mean number of events, and functions thereof (e.g., differences and standardized means) with accompanying confidence intervals.

This paper aims to introduce an appropriate parametric estimator of the mean number of events in the presence of competing risks using flexible parametric survival models (FPMs). The paper is organized as follows: We start by introducing necessary definitions and subsequently present the existing nonparametric estimator of Cook and Lawless and our parametric estimator based on FPMs in Section 2. Section 3 shows results of a simulation study to test the validity of the proposed estimator. Section 4 includes an illustrative example of the proposed estimator using data from a study on hospital readmissions (available in the frailtypack package for R). Finally, we discuss our proposed method and suggest some possible extensions in Section 5.

## 2 Methods

**FIGURE 1:**
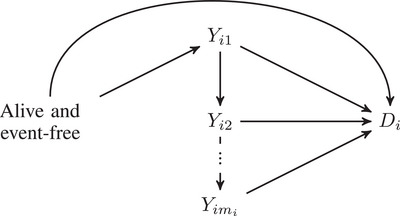
Illustration of the event process where the nodes symbolize different states of the event process.

We follow the notation introduced in Rondeau (2010). For each i=1,⋯,n, and $j=1,\cdots ,m_{i}$, we denote by $Y_{ij}$ the jth recurrent event time for the ith individual. In addition, we let $D_{i}$ be the competing event time, and $C_{i}$ the time of right‐censoring. Each observation time is represented by $T_{ij}=\min\left(Y_{ij},D_{i},C_{i}\right)$ and the maximum follow‐up for each individual by $T_{i}=\min\left(D_{i},C_{i}\right)$. We further define event indicators for both types of event as $\delta_{ij}=I_{\left\{T_{ij}=Y_{ij}\right\}}$ for the recurrent event process and $\delta_{i}=I_{\left\{T_{i}=D_{i}\right\}}$ for the competing event. Note that within the same individual we might observe several event times, one for each occurrence of the recurrent event ($T_{ij}$), and additionally the time of competing event or censoring ($T_{i}$). After experiencing a competing event, an individual cannot experience any other event. Figure 1 shows an illustration of the assumed underlying event process. We define the hazard function for the competing event as

(1) $$h\left(t_{i}\right)=\frac{d}{dt_{i}}H\left(t_{i}\right)=\lim_{\Delta t_{i}\to 0}\frac{P(t_{i}\leq T_{i}<t_{i}+\Delta t_{i}|T_{i}\geq t_{i})}{\Delta t_{i}},$$

which gives the instantaneous probability of experiencing the competing event conditioning on being event‐free up until time $t_{i}$. The associated survival function $S(t_{i})$ gives the probability of being free of the competing event (in most cases being alive) until time $t_{i}$:

(2) $$S\left(t_{i}\right)=P\left(T_{i}>t_{i}\right).$$

For the recurrent event, we instead define a memoryless (i.e., not dependent on the event history) intensity function

(3) $$\lambda \left(t_{ij}\right)=\frac{d}{dt_{ij}}\Lambda \left(t_{ij}\right)=\lim_{\Delta t_{ij}\to 0}\frac{P(t_{ij}\leq T_{ij}<t_{ij}+\Delta t_{ij})}{\Delta t_{ij}},$$

which is similar to the hazard function except that it is not conditioning on being event‐free up until $t_{ij}$. This implies that individuals remain in the risk set also after experiencing one or several occurrences of the recurrent event.

### 2.1 Mean Number of Events Function

We can now introduce a function for the mean number of events up to time t, E[N(t)], in the presence of competing events based on the survival function for the competing event S(t) and the intensity function of the recurrent event λ(t) following the estimator suggested by Cook and Lawless (1997).

(4) $$\mu \left(t\right)=E\left[N\left(t\right)\right]=\int_{0}^{t}S\left(u\right)\lambda \left(u\right)du.$$

Cook and Lawless used a nonparametric (Kaplan–Meier) estimator of S(t) and the Nelson–Aalen estimator of the cumulative hazard function incorporating recurrent events (Cook and Lawless 1997; Nelson 1969). Asymptotic properties of this estimator have been discussed by Ghosh and Lin (2000).

### 2.2 Flexible Parametric Survival Models

FPMs are a class of survival models, mostly estimated on the log cumulative hazard scale, that use a spline function for flexibly modeling the baseline cumulative hazard function. We will briefly introduce FPMs before showing how they can be used to estimate the mean number of events with competing events present.

For introducing FPMs, let us assume the following simple Weibull model for the distribution of event times:

(5) $$S\left(t\right)=\exp\left[-\kappa t^{\gamma_{1}}\right],$$

where κ, and $\gamma_{1}$ are the scale and shape parameter of the Weibull distribution, respectively. Transforming Equation (5) to the log cumulative hazard scale shows

(6) $$\begin{array}{ccc} H(t) & = & -\log\left[S\left(t\right)\right]=\kappa t^{\gamma_{1}}\log\left[H\left(t\right)\right]\\ & & =\log\left[\kappa \right]+\gamma_{1}\log\left[t\right]=\gamma_{0}+\gamma_{1}\log\left[t\right]. \end{array}$$

In FPMs, the baseline cumulative hazard function is commonly modeled using a restricted cubic spline function of log(t) with a vector of knots l and parameters γ. Knots are commonly placed at the minimum and maximum value of log(t) and evenly distributed across the distribution of log(t) depending on the number of knots specified (Durrleman and Simon 1989).

(7) log[H(t)]=s[log(t)|γ,l].

Adding a vector of covariates x with associated coefficients β to the model for the cumulative hazard function yield the following standard definition of FPM:

(8) $$\log\left[H\left(t|x\right)\right]=\log\left[H_{0}\left(t\right)\right]+x^{T}\beta ,$$

where $H_{0}\left(t\right)$ denotes the cumulative baseline hazard function.

We can then obtain the survival function and hazard function from the log cumulative hazard function

(9) S(t)=exp{−exp[logH(t)]},

(10) $$h\left(t|x\right)=\frac{ds[\log(t)|\gamma ,l]}{dt}\exp\left[\log H\left(t|x\right)\right].$$

We propose that estimates of S(t) and λ(t) obtained from an FPM can subsequently be used to obtain fully parametric estimates of the mean number of events (E[N(t)]).

Compared to the Cox model, FPMs rely on a parametric specification of the baseline cumulative hazard function as well as covariate effects, which facilitates easy estimation of various transformations of the hazard function with accompanying confidence intervals. This is especially useful when combining different transformations of the hazard and intensity functions in order to estimate the mean number of events function.

### 2.3 A Parametric Estimator of the Mean Number of Events

Hinchliffe and Lambert (2013) showed that FPMs can be used to estimate the cause‐specific CIF by simultaneously estimating multiple outcomes with the same FPM. A similar approach can be used for the estimation of E[N(t)]. For this, we introduce a joint time‐to‐first‐event and Andersen–Gill‐type FPM. For the Andersen–Gill‐type FPM estimation, every event episode is included in the risk set (Royston and Lambert 2011). Follow‐up ends at the time of censoring or experiencing the competing event ($T_{i}$). Thus, the risk set at time t will consist of all individuals that have not yet experienced a competing event or been censored. We suggest the following forms for the log cumulative hazard function of the recurrent event and the competing event:

(11) $$\begin{array}{cc} \log\Lambda_{ij}\left(t|x_{1ij}\right) & =s\left[\log\left(t\right)|\gamma_{1},l_{1}\right]+x_{1ij}^{T}\beta_{1},\\ \log H_{i}\left(t|x_{2i}\right) & =s\left[\log\left(t\right)|\gamma_{2},l_{2}\right]+x_{2i}^{T}\beta_{2}, \end{array}$$

where $x_{1}$ and $x_{2}$ are vectors of covariates used for modeling the recurrent event and competing event, respectively, with associated vectors of coefficients $\beta_{1}$ and $\beta_{2}$. Similarly, $\gamma_{1}$, $\gamma_{2}$, $l_{1}$, $l_{2}$ are vectors of parameters and knots for the spline functions of the baseline cumulative hazards. Note that $x_{1}$ and $x_{2}$ can overlap if the same covariate is assumed to have an effect on both event types. Equation (11) can be further extended by including time‐varying effects:

(12) $$\begin{array}{ccc} \log\Lambda_{ij}\left(t|x_{1ij}\right) & = & s\left[\log\left(t\right)|\gamma_{1},l_{10}\right]+\sum_{v=1}^{V_{1}}s\left[\log\left(t\right)|\omega_{1v},l_{1v}\right]x_{1ijv}\\ & & +\;x_{1ij}^{T}\beta_{1},\log H_{i}\left(t|x_{2i}\right)=s\left[\log\left(t\right)|\gamma_{2},l_{20}\right]\\ & & +\sum_{v=1}^{V_{2}}s\left[\log\left(t\right)|\omega_{2v},l_{2v}\right]x_{2iv}+x_{2i}^{T}\beta_{2}, \end{array}$$

where $l_{1v}$, $l_{2v}$, $\omega_{1v}$, $\omega_{2v}$, and $V_{1}$, $V_{2}$ are the knots positions, associated parameters for the vth time‐varying effect, and the number of time‐varying effects in the recurrent and competing event model, respectively.

Each individual i contribution to the likelihood can be split into two parts, where one contribution is to the likelihood of the recurrent event model $L_{1i}$ and the other to the likelihood of the competing event model $L_{2i}$ assuming that the recurrent and competing event processes are independent. We will revisit this assumption further down at the end of this section. The log‐likelihood contribution of individual i for a parametric counting process model can be defined as

(13) $$\begin{array}{ccc} \log L_{1i}\left(\theta_{1}|x_{1ij},t_{ij}\right) & = & \sum_{j=1}^{m_{i}}\delta_{ij}\log\left[\lambda \left(t_{ij}|x_{1ij}\right)\right]-\Lambda \left(t_{ij}|x_{1ij}\right)\\ & & +\;\Lambda (t_{ij-1}|x_{1ij}), \end{array}$$

following the definition by Borgan (1984), with $\theta_{1}=\left(\beta_{1},\gamma_{1}\right)$.

The contribution of the ith individual to the log‐likelihood for a standard parametric survival model is defined as

(14) $$\log L_{2i}\left(\theta_{2}|x_{2i},t_{i}\right)=\delta_{i}\log\left[h\left(t_{i}|x_{2i}\right)\right]+\log\left[S\left(t_{i}|x_{2i}\right)\right],$$

where $\theta_{2}=\left(\beta_{2},\gamma_{2}\right)$. Substituting S(t) with H(t) yields

(15) $$\log L_{2i}\left(\theta_{2}|x_{2i},t_{i}\right)=\delta_{i}\log\left[h\left(t_{i}|x_{2i}\right)\right]-H\left(t_{i}|x_{2i}\right).$$

Combining Equations (13) and (15) yields the contribution of the ith individual to the log‐likelihood of the joint model for the recurrent and competing event processes.

(16) $$\begin{array}{ccc} & & \log L_{i}\left(\theta |x_{i},t_{ij},t_{i}\right)=\log L_{1i}\left(\theta_{1}|x_{1ij},t_{ij}\right)+\log L_{2i}\left(\theta_{2}|x_{2i},t_{i}\right)\\ & & =\sum_{j=1}^{m_{i}}\{\delta_{ij}\log\left[\lambda \left(t_{ij}|x_{1ij}\right)\right]-\Lambda \left(t_{ij}|x_{1ij}\right)+\Lambda \left(t_{ij-1}|x_{1ij}\right)\}\\ & & +\;\delta_{i}\log\left[h\left(t_{i}|x_{2i}\right)\right]-H\left(t_{i}|x_{2i}\right). \end{array}$$

Table 1 shows the data setup used for calculating the likelihood contribution of the ith individual for the joint model in Equation (16). The survivor function, $S(t_{i})$, is estimated from the model above using the relation in Equation (9). Similarly, the intensity function for the recurrent event is estimated using the relation in Equation (10), but exchanging the hazard with the intensity function

(17) $$\hat{\lambda }\left(t_{ij}|x_{1}\right)=\frac{ds[\log\left(t_{ij}\right)|\gamma_{1},l_{1}]}{dt_{ij}}\exp\left[\log\Lambda \left(t_{ij}|x_{1}\right)\right].$$

**TABLE 1:** Data setup for the joint likelihood in Equation (16). *t.start*: time at the start of follow‐up; *t.stop* is time at the end of follow‐up; *re*: indicator for rows contributing to $L_{1}$; *ce*: indicator for rows contributing to $L_{2}$; x: a vector of covariates for modeling the intensity function of the recurrent ($x_{1}$) and the competing events ($x_{2}$).

*id*	*t.start*	*t*.*stop*	δ	*ce*	*re*	x
i	0	$t_{i1}$	$\delta_{i1}$	0	1	$x_{1i1}$
i	$t_{i1}$	$t_{i2}$	$\delta_{i2}$	0	1	$x_{1i2}$
i	$t_{ij-1}$	$t_{ij}$	$\delta_{ij}$	0	1	$x_{1ij}$
⋮	⋮	⋮	⋮	⋮	⋮	⋮
i	$t_{im_{i}-1}$	$t_{im_{i}}$	$\delta_{im_{i}}$	0	1	$x_{1im_{i}}$
i	0	$t_{i}$	$\delta_{i}$	1	0	$x_{2i}$

Using the parametric estimates from the models above, we can now calculate the mean number of events at time t using Equation (4). Estimating E[N(t)] requires the integration of the product of S(t) and λ(t) over t. We suggest using Romberg's method to approximate the integral (Romberg 1955):

(18) $$\begin{array}{ccc} \hat{\mu }\left(t\right) & = & \int_{0}^{t}\hat{S}\left(u\right)\hat{\lambda }\left(u\right)du=\int_{0}^{t}g\left(u\right)du\\ & & =R_{a,b}\left(0,t\right)+O\left(s_{a}^{b+1}\right), \end{array}$$

where $R_{a,b}\left(0,t\right)$ is an approximation of the integral from 0 to t using Romberg's method with a∈{1,2,3,⋯} steps, b∈{1,2,3,⋯} extrapolations, and an error of magnitude $O(s_{a}^{b+1})$ with step size $s_{a}=t/2^{a}$ (Romberg 1955). Romberg's method includes two steps. First, the integral is approximated using the composite Trapezoidal rule with $2^{a}$ steps of size $s_{a}$. Second, the estimates of the Trapezoidal rule are combined using Richardson's extrapolation to increase the accuracy of the approximation. This extrapolation is then continued b times based on the previous extrapolations. The general form of Romberg's method for estimating the mean number of events in our model is

(19) $$R_{a,b}\left(0,t\right)=\left\{\begin{array}{cc} \frac{s_{a}}{2}\left[g\left(0\right)+2\sum_{j=1}^{2^{a-1}}g\left(u_{j}\right)+g\left(t\right)\right], & \;\text{if}\;b=1,\\ R_{a,b}\left(0,t\right)+\frac{R_{a,b-1}\left(0,t\right)-R_{a-1,b-1}\left(0,t\right)}{4^{k-1}-1}, & \;\text{if}\;b>1, \end{array}\right.$$

where $u_{j}=j\times s_{a}$ are the middle points considered for the composite Trapezoidal rule. We propose to use b=5 extrapolations and test an increasing number of steps until convergence is reached, that is, the estimate of the integral is stable when adding one more extrapolation step.

Lastly, the intraobservation dependence between events within the same individual, requires an adjustment of the variance estimate. We suggest using a cluster‐based robust variance estimator $V_{r}$ as described by Liu, Pawitan, and Clements (2017) for accounting for this intraobservation dependence:

(20) $$V_{r}=\hat{V}\sum_{i=1}^{n}\left[{\left(\sum_{j=1}^{m_{i}}w_{ij}\right)}^{T}\left(\sum_{j=1}^{m_{i}}w_{ij}\right)\right]\hat{V},$$

where $\hat{V}$ is the observed covariance matrix for the full expression in Equation (16), and $w_{ij}=d\log L_{ij}/d\theta$. Confidence intervals for $\hat{\mu }\left(t\right)$ can subsequently be estimated based on the robust variance estimates using the delta method.

### 2.4 Implementation

The proposed method is implemented in the R‐package JointFPM which is available on CRAN (https://cran.r‐project.org/web/packages/JointFPM). The package includes one function for jointly modeling the competing and recurrent events using an FPM, and one postestimation function for estimating conditional and marginal estimates of the mean number of events at different time points, as well as differences in mean number of events between two groups. The package utilizes the rstpm2 package for fitting the FPM (Liu, Pawitan, and Clements 2017).

## 3 Simulation Study

### 3.1 Aim

This simulation study aimed to assess the bias and coverage of our parametric estimator of μ(t) under different scenarios altering both the rate of the competing and the recurrent event including situations in which both the rates of the recurrent and the competing events are low and very high. We used the ADEMP framework suggested by Morris, White, and Crowther (2019) for planning and performing our simulation study.

### 3.2 Data Generation Mechanisms

We used the simrec package for jointly simulating the intensity processes of the competing event $h(t_{i})$ and the recurrent event $\lambda (t_{ij})$ (Jahn‐Eimermacher et al. 2015):

(21) $$\left\{\begin{array}{cc} & \lambda \left(t_{ij}|x\right)=\lambda_{0}\left(t_{ij}\right)\times \exp\left(x\beta_{1}\right)\\ & h\left(t_{i}|x\right)=h_{0}\left(t_{i}\right)\times \exp\left(x\beta_{2}\right), \end{array}\right.$$

where $\lambda_{0}\left(t_{ij}\right)$ and $h_{0}\left(t_{i}\right)$ denote the baseline intensity and hazard function for the recurrent and competing event processes, which are assumed to follow a Weibull distribution with scale parameters ρ and ι, and shape parameters ϕ and ψ, respectively:

(22) $$\begin{array}{cc} T_{ij} & ∼\text{Weibull}(\rho ,\phi )\\ T_{i} & ∼\text{Weibull}(\iota ,\psi ). \end{array}$$

**FIGURE 2:**
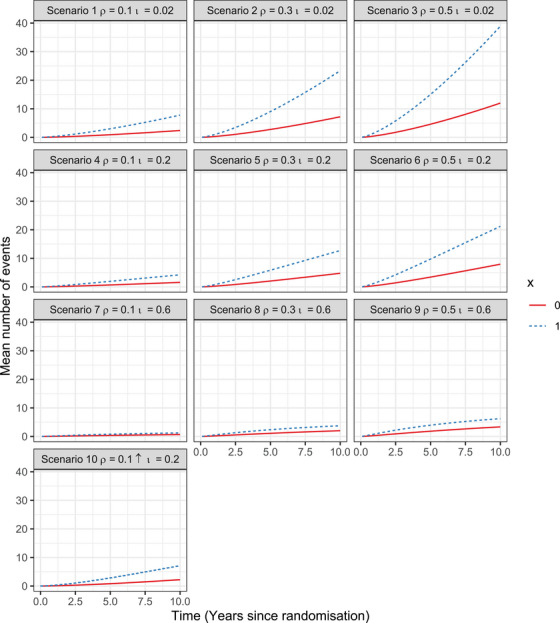
Estimates of the mean number of events in the simulated populations of 10 million individuals across time for scenarios 1 to 10.

We simulated a study population of 10 million individuals, who were assigned to either $x_{1}=1$ or $x_{1}=0$ following a binomial distribution with mean equal to 0.5. The effect of $x_{1}$ on the intensity function for the competing and recurrent events was set to $\beta_{11}=1.2$ and $\beta_{21}=0.4$, respectively. In a supplementary analysis based on scenario 5, we additionally included a continuous variable $x_{2}$ which followed a standard normal distribution. The effect of $x_{2}$ on the competing and recurrent event processes was set to $\beta_{12}=1.1$ and $\beta_{22}=0.8$, respectively. For illustrative purposes, we included the same covariate x in both the competing and recurrent event functions, but with different effect size. However, as shown in Equation (11), $x_{1}$ and $x_{2}$ may also include different covariates. All individuals were followed from the time of randomization (t=0) until the occurrence of the competing event or a maximum follow‐up of 10 years. The scenarios considered for the simulation are presented in Table 2. In scenarios 1 to 9, we considered the event rate to be constant across all event episodes. We relaxed this assumption in scenario 10 and instead assumed an event rate function that increased with the number of previous events. Figure 2 shows the true underlying mean number of events in the different scenarios.

**TABLE 2:** Selected distribution parameters for the recurrent and competing event distributions in different simulation scenarios. Parameters ψ=0.5 and ϕ=1.4 throughout all simulations.

Scenario	ρ	(Event rate)	ι	(Survival)
1	0.1	(low)	0.02	(high)
2	0.3	(medium)	0.02	(high)
3	0.5	(high)	0.02	(high)
4	0.1	(low)	0.2	(medium)
5	0.3	(medium)	0.2	(medium)
6	0.5	(high)	0.2	(medium)
7	0.1	(low)	0.6	(low)
8	0.3	(medium)	0.6	(low)
9	0.5	(high)	0.6	(low)
10	$0.1\times (1+\frac{j}{j+1})$	(low, increasing)	0.2	(medium)

### 3.3 Estimands

The target of the simulations was the estimated mean number of events at 2.5, 5, and 10 years. In a supplementary analysis, we additionally targeted estimates of the difference in the mean number of events at 2.5, 5, and 10 years (μ(t|x=0)−μ(t|x=1)).

### 3.4 Method

We compared the analytical expectation of the mean number of events to our parametric estimator of μ(t) except for scenario 10, in which we instead calculated the mean number of events in the full data of $10^{6}$ observations and used that as benchmark. A detailed description of the benchmark estimates can be found in Supporting Information Section A.1. For testing our parametric model, we sampled n=1000 individuals from the full data 1900 times, with $10^{6}$ observations and estimated μ(t) at 2.5, 5, and 10 years in each sample. The number of repetitions was derived based on the expected coverage of $\hat{\mu }\left(t\right)$ of 95% in order to limit the Monte Carlo standard error to 0.5 (Morris, White, and Crowther 2019). The limit of the Monte Carlo error was set at 0.5 in order to detect deviations from the expected coverage by 1 percentage point at the α=0.05 significance threshold. In a supplementary analysis, we compared the calculated differences in the mean number of events comparing observations with x=0 and those with x=1 to our parametric estimator μ(t|x=0)−μ(t|x=1). In addition, we also compared an estimate of the mean number of events when ignoring the competing event, that is, Λ(t) with our benchmark estimates as well as the nonparametric estimator of the mean number of events introduced by Cook and Lawless.

### 3.5 Performance Measures

Estimates of absolute bias and coverage are reported to assess the performance of the described parametric estimator of μ(t) (Morris, White, and Crowther 2019). Due to the differences in the range of estimates across the different scenarios, we additionally provide estimates of relative bias ($E\{\hat{\mu }\left(t\right)-\mu \left(t\right)\}/\mu \left(t\right)$) to assess performance. We evaluated differences between the observed and expected performance measures using 95% confidence intervals constructed based on Monte Carlo standard error for each performance measure. Bias, relative bias, and coverage were reported to be significantly different when the confidence interval did not include 0, 0%, or 95%, respectively.

### 3.6 Simulation Results

**FIGURE 3:**
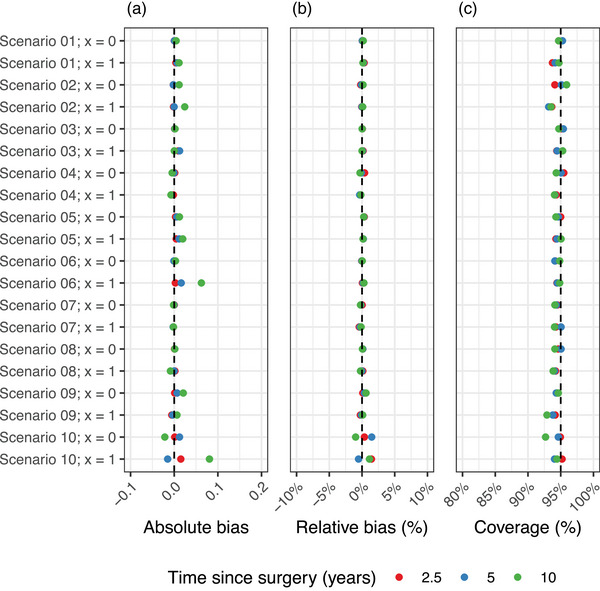
Estimates of absolute bias (A), relative bias (B), and coverage (C) across the different simulation scenarios for both groups with x=0, and x=1.

The simulation study showed overall low bias and good coverage across the different scenarios and time points except for scenario 10 (Table 3, Figure 3). The average absolute and relative bias was less than 0.01 and 0.5% in most scenarios, respectively. Coverage was above 94% and not significantly different to the expected 95% in most of the scenarios. However, point estimates in scenario 10 were more biased than in the other scenarios, likely due to the added change of the recurrent event rate based on the number of previous events. The highest relative bias was observed for the 5‐year estimate in the exposed group in scenario 10 (1.476%). We found that the coverage was overall high for estimates in scenario 10.

**TABLE 3:** Estimates of bias, relative bias, and coverage at 2.5, 5, and 10 years of μ(t). Estimates highlighted in bold font were statistically significantly different from their expected value at the 5% threshold based on their Monte Carlo standard errors. Expected values for bias, relative bias, and coverage were 0, 0%, and 95%, respectively.

		At 2.5 years			At 5 years			At 10 years		
Scenario	X	Bias	Rel. bias	Coverage	Bias	Rel. bias	Coverage	Bias	Rel. bias	Coverage
**1**	0	0.000	0.038%	94.7%	0.000	0.052%	95.3%	0.004	0.183%	94.7%
	1	**0.004**	**0.327%**	**93.7%**	**0.006**	**0.213%**	94.2%	**0.011**	**0.142%**	94.7%
**2**	0	**−0.002**	**−0.204%**	94.1%	−0.002	−0.072%	95.1%	**0.011**	**0.157%**	**95.9%**
	1	−0.002	−0.046%	**93.6%**	0.000	−0.003%	**93.2%**	**0.024**	**0.103%**	**93.5%**
**3**	0	0.001	0.050%	95.1%	0.001	0.027%	95.4%	0.002	0.016%	94.7%
	1	**0.009**	**0.162%**	94.4%	**0.012**	**0.082%**	94.5%	0.000	0.000%	95.3%
**4**	0	**0.001**	**0.391%**	95.5%	−0.001	−0.109%	95.1%	**−0.005**	**−0.305%**	94.3%
	1	**−0.002**	**−0.236%**	94.3%	**−0.007**	**−0.355%**	94.1%	−0.008	−0.177%	94.1%
**5**	0	**0.003**	**0.344%**	95.0%	**0.006**	**0.282%**	94.6%	**0.012**	**0.250%**	94.3%
	1	**0.005**	**0.178%**	94.3%	**0.011**	**0.196%**	94.4%	0.020	0.154%	95.1%
**6**	0	−0.001	−0.051%	94.2%	−0.001	−0.024%	94.1%	0.003	0.037%	94.8%
	1	0.003	0.060%	94.4%	**0.016**	**0.164%**	94.5%	**0.062**	**0.293%**	94.8%
**7**	0	0.000	0.023%	94.5%	−0.001	−0.158%	94.3%	−0.002	−0.232%	94.1%
	1	**−0.002**	**−0.428%**	94.3%	−0.002	−0.300%	95.1%	−0.002	−0.141%	94.1%
**8**	0	0.001	0.121%	94.6%	0.001	0.116%	95.1%	0.001	0.065%	94.1%
	1	0.002	0.120%	94.2%	0.000	−0.016%	94.1%	−0.009	−0.233%	**93.8%**
**9**	0	0.001	0.118%	94.5%	**0.007**	**0.361%**	94.3%	**0.021**	**0.616%**	94.6%
	1	**−0.006**	**−0.258%**	94.2%	−0.003	−0.088%	**93.8%**	0.006	0.101%	**92.9%**
**10**	0	0.001	0.367%	94.9%	**0.012**	**1.476%**	94.6%	**−0.022**	**−0.977%**	**92.7%**
	1	**0.015**	**1.438%**	95.2%	**−0.015**	**−0.531%**	94.1%	**0.081**	**1.138%**	94.4%

The supplementary analysis of scenario 5 with addition of a continuous variable showed low bias and good coverage within the range of observed values of $x_{2}$ (Table S1). Extrapolations of the mean number of events outside the range of observed values ($x_{2}=3$) were biased.

We also performed a supplementary analysis of estimates of the differences in the mean number of events, which showed low bias and good coverage across the scenarios (Table S2). Similar to Table 3, point estimates of the difference in the mean number of events as well as coverage was lower in scenario 10.

A comparison of the simulation results of the parametric estimate with those of the nonparametric estimator of the mean number of events, presented in Table S3, showed overall similar results. However, the parametric estimator performed better than the nonparametric estimator with regards to bias and coverage when estimating the mean number of events at the end of follow‐up, that is, at 10 years.

Comparing estimates of the mean number of events with estimates of the cumulative intensity function (Λ(t)), that is, ignoring the competing events, showed that ignoring the competing event let to highly biased estimates of the mean number of events (Table S4). The bias was, as expected, especially high in scenarios with low survival, that is, scenarios 7 to 9. The relative bias reached up to 570% at 10 years in scenario 9.

## 4 Readmission After Colon Cancer Surgery

We use data from a single‐center cohort study on sex differences in hospital readmissions after colon cancer surgery previously described by González et al. (2005) and available as part of the frailtypack package in R. The data include information on 239 female and 164 male patients that underwent surgery for either colon or rectal cancer in the Hospital de Bellvitge in Spain between January 1996 and December 1998. Each patient was followed for hospital readmissions related to colorectal cancer from the time of surgery until emigration, change of hospital, death, or June 2002, whichever came first.

In their original analysis, the authors presented plots of the probability of (first) rehospitalization after colorectal cancer surgery across time since surgery for both males and females stratified by whether they died during the time of follow‐up. Due to the recurrent nature of hospital readmissions, we instead estimated the mean number of rehospitalizations after surgery in the presence of death as a competing event for both males and females by adjuvant chemotherapy treatment status using our suggested method. For comparison, we also applied the nonparametric estimator suggested by Cook and Lawless (cf. Section 2.1).

We restricted our analysis to a maximum follow‐up of 1500 days in order to balance differences in follow‐up times across chemotherapy treatment groups. Both our model for the recurrent event of hospital readmissions and for the competing event of death included time‐varying effects for sex ($x_{FM}$) and chemotherapy treatment status ($x_{CE}$), as well as an interaction term between these two variables. Chemotherapy treatment status was coded as 1 (received chemotherapy after surgery), and 0 (did not receive chemotherapy after surgery). Sex was included in the model using an indicator for females (Yes/No).

(23) $$\begin{array}{cc} \log\{\Lambda (t|x)\}= & s[\log\left(t\right)|\gamma_{1},l_{10}]+\\ & s\left[\log\left(t\right)|\omega_{11},l_{11}\right]x_{\text{FM}}+\\ & s\left[\log\left(t\right)|\omega_{12},l_{12}\right]x_{\text{CE}}+\\ & \beta_{11}x_{\text{FM}}+\beta_{12}x_{\text{CE}}+\beta_{13}x_{\text{FM}}x_{\text{CE}}\\ \log\{H(t|x)\}= & s[\log\left(t\right)|\gamma_{2},l_{20}]+\\ & s\left[\log\left(t\right)|\omega_{21},l_{21}\right]x_{\text{FM}}+\\ & s\left[\log\left(t\right)|\omega_{22},l_{22}\right]x_{\text{CE}}+\\ & \beta_{21}x_{\text{FM}}+\beta_{22}x_{\text{CE}}+\beta_{23}x_{\text{FM}}x_{\text{CE}}. \end{array}$$

Before jointly modeling the competing and recurrent event processes, the data used for the analysis need to be prepared in a stacked long format in which each individual has one row for the competing event and one row for each risk episode of the recurrent event, that is, every individual is followed from the initial start of follow‐up until the first occurrence of the event of interest, and then for all subsequent events, from the time of the previous one until the occurrence of the next. Each row requires a column each for the start of follow‐up, the end of follow‐up, the event type, and an event indicator. For the competing event, the follow‐up starts at time 0 and ends at time $t_{i}$. For the recurrent event, the follow‐up for the first risk episode starts at time 0 and ends at time $t_{i1}$. The follow‐up for subsequent events j starts at time $t_{i,j-1}$ and end at time $t_{ij}$. Table 4 shows the stacked data format for the data used in this example.

**TABLE 4:** Example of a data set in stacked long format for the first three individuals in the readmission data set included in the *frailtypack* package for R. *t.start*: time at the start of follow‐up; *t.stop* is time at the end of follow‐up; *event*: overall event indicator for both the competing and recurrent events; *ce*: indicator for rows used to model the competing risk; *re*: indicator for rows used to model the competing risk; *chemo*: chemotherapy treatment indicator; *female*: indicator for being female.

id	t.start	t.stop	event	ce	re	chemo	female
1	0	24	1	0	1	1	1
1	24	457	1	0	1	1	1
1	457	1037	0	0	1	1	1
1	0	1037	0	1	0	1	1
2	0	489	1	0	1	0	0
2	489	1182	0	0	1	0	0
2	0	1182	0	1	0	0	0
3	0	15	1	0	1	0	0
3	15	783	0	0	1	0	0
3	0	783	1	1	0	0	0

**FIGURE 4:**
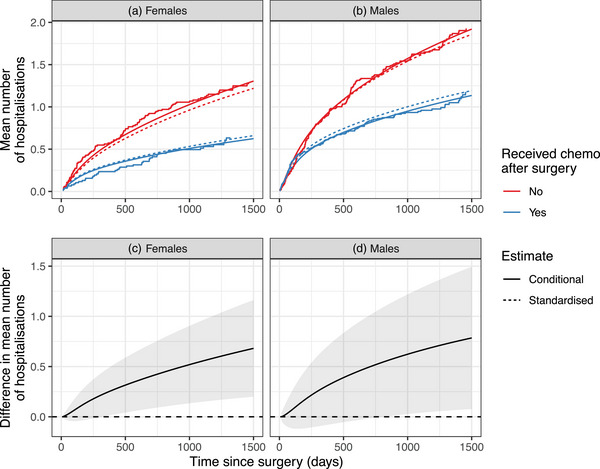
Mean number of rehospitalizations after surgery for colon cancer, by sex and chemotherapy treatment. The upper panels show the conditional and standardized mean number of hospitalizations, for females (A) and males (B). The smooth curve represents our parametric estimator using flexible parametric models, and the step function the Gosh and Lin estimator. The lower panels (C–D) show the difference in the mean number of rehospitalizations with 95% confidence intervals comparing patients receiving chemotherapy to those who did not receive chemotherapy after colon cancer surgery for females and males.

Different combinations of degrees of freedom (dfs) for the baseline hazard function for the death and the hospital readmission as well as for time‐varying effects of sex and chemotherapy status on both events were evaluated. The best model fit based on Akaike information criterion (AIC) was obtained for a model with 2 dfs for both the baseline hazard and baseline intensity functions and 2 dfs for modeling the time‐varying effect of sex and chemotherapy status on hospital readmission, whereas 2 and 1 df showed the best fit for the time‐varying effect of sex and chemotherapy treatment on death, respectively. Based on this model, we estimated the mean number of hospital readmissions and the difference in hospital readmissions comparing patients who received chemotherapy after surgery to those who did not (Figure 4). In addition, we added Charlson Comorbidity Index and colon cancer stage at the time of surgery to the model in Equation (23) in order to estimate standardized estimates of the mean number of events as well as standardized differences.

As seen in Figure 4, colorectal cancer patients who underwent chemotherapy treatment after colorectal cancer surgery experienced lower mean numbers of rehospitalizations, among both females and males. The estimated mean number of hospital readmissions was 0.62 (95% CI: 0.40–0.85) and 1.14 (95% CI: 0.83–1.44) after 1500 days postsurgery for females and males who received chemotherapy treatment after surgery, and 1.31 (95% CI: 0.88–1.74) and 1.92 (95% CI: 1.29–2.55) for females and males who did not receive chemotherapy after surgery. After additional adjustment for comorbidity and colon cancer stage at time of surgery, the standardized mean number of hospitalizations after surgery among patients that received chemotherapy and those that did not was slightly smaller than the nonstandardized estimates. The standardized differences for females and males were 0.66 (95% CI: 0.03–1.23), and 0.55 (95% CI: 0.10–1.02), respectively. Figures S1 and S2 show the survival and cumulative hazard function of rehospitalization after colon cancer surgery, respectively. The higher survival and readmission rate among females not receiving chemotherapy after treatment suggests that the higher mean number of hospital readmissions in this group is driven by both processes. Whereas the higher readmission rate among males who did not receive adjuvant chemotherapy seems to be mainly driven by their higher hospitalization rate when compared to those males who did receive such treatment, as males not treated with chemotherapy after surgery had poorer survival than those who were.

Ignoring the competing event would lead to an overestimation of the mean number of rehospitalizations as individuals who died are still considered to be at risk for rehospitalization. Figure S3 shows a comparison of the mean number of rehospitalizations and the cumulative intensity function of rehospitalizations. The difference in both estimates is most prevalent when comparing absolute estimates of the mean number of rehospitalizations. The effect on estimates of the difference in the mean number of rehospitalizations is less pronounced.

## 5 Discussion

We have developed and evaluated a parametric estimator of the mean number of recurrent events in the presence of competing events. Our simulations revealed overall low bias and good coverage of our estimator. We implemented our method in the R package JointFPM which is readily available on CRAN. Lastly, we showcased our method using an example of hospital readmissions after colon cancer surgery. In this illustrative example, our suggested estimator facilitated clear and interpretable comparisons between the two treatment groups through the estimation of differences in the mean number of events together with confidence intervals.

From a causal perspective, the mean number of events estimates the total effect of the exposure variable on the outcome of interest which might be mediated through effects of the exposure on previous events (Jahn‐Eimermacher et al. 2017). This total effect estimate can be used to quantify overall disease burden or disease intensity. However, if the interest lies in episode‐specific transition rates, the use of other models, for example, multistate models, might be more appropriate. However, in these cases, estimates of the mean number of events might still be a useful augmentation of the main analysis. In contrast to our marginal model, partially conditional models such as multistate models estimate the direct effect of the exposure on the outcome, that is, the effect estimate does not include effects mediated through an effect on previous event rates (Jahn‐Eimermacher et al. 2017).

As a strength of our approach, the fully parametric model allows for straightforward postestimation of the mean number of events and differences thereof together with confidence interval estimated using the delta method. Importantly, the parametric approach allows the modeling of continuous variables as well as the inclusion of time‐varying effects. Currently, our implementation supports the prediction of conditional and marginal estimates of the mean number of events and differences thereof. However, other transformations of interest, for example, ratios, can be added in the future. Furthermore, our method does not require separate models for each transition from between recurrent events nor does it require prespecifying the maximum number of occurrences that can be included in the model which would be necessary when using a multistate modeling approach. Possible interesting future extension of our approach includes the incorporation of multiple competing events and interval censored event times.

As a potential limitation, we currently fit the model assuming an independence likelihood and then estimate the variance–covariance matrix using a sandwich estimator (Liu, Pawitan, and Clements 2017). An alternative approach would be to estimate a frailty term which could be a potential future extension, which could also be used for allowing the competing event rate to depend on the number of previous occurrences of the recurrent event (Liu, Pawitan, and Clements 2017; Chauvet and Rondeau 2023). Also, we assume that the recurrent event has a clock‐forward (Markov) time scale. Extending the model to a clock‐reset time scale could be a future generalization of the model. In our simulations, we only considered Weibull distributions for both the hazard and the intensity functions (for the competing and recurrent events, respectively). The aim of our simulation study was to assess the validity of our proposed method in general rather than testing the goodness of fit under different baseline hazard shapes, as FPMs have previously been validated for a variety of baseline hazard shapes (Liu, Pawitan, and Clements 2018). To assess the model fit, we suggest using AIC or the Bayesian information criterion as guidance. We found our model to be biased in the scenario with nonconstant recurrent‐event rates across event episodes. Using methods that allow for episode‐specific recurrent‐event rates, such as multistate models or the Prentice–Williams–Petersen models, might be more reliable in these situations.

In conclusion, easy estimation of the mean number of events in the presence of competing risks and contrasts thereof is highly relevant in a variety of medical research areas including, for example, population‐based cancer studies or epidemiology of cardiovascular disease. Only focusing on the time to first event in this situation disregards important information about the disease process. Our implementation of a parametric estimator of the mean number of events offers a valuable complement to existing methods in time‐to‐event analyses, and can indeed be useful when the interest lies in summarizing a recurrent event process while taking competing events into account.

## Conflicts of Interest

The authors declare no conflicts of interest.

### Open Research Badges

This article has earned an Open Data badge for making publicly available the digitally‐shareable data necessary to reproduce the reported results. The data is available in the Supporting Information section.

This article has earned an open data badge “**Reproducible Research**” for making publicly available the code necessary to reproduce the reported results. The results reported in this article could fully be reproduced.

## Supporting information

Supporting Information

## Data Availability

R source code for the simulation study as well as for the illustrative example is openly available on GitHub at https://github.com/entjos/JointFPM_simulation_study.
