# Supplementary material for: Parametric Estimation of the Mean Number of Events in the Presence of Competing Risks
Source: Biom J. 2025 Feb 18;67(1):e70038. doi: 10.1002/bimj.70038 (PMC11836554; doi:10.1002/bimj.70038)
Supplement: Supplementary file 1 — Supporting Information [file BIMJ-67-e70038-s001.pdf]

APPENDIX
PARAMETRIC ESTIMATION OF THE MEAN NUMBER OF EVENTS IN THE PRESENCE OF COMPETING RISKS

Joshua P. Entrop *
Clinical Epidemiology Division,
Department of Medicine, Solna,
Karolinska Institutet,
Stockholm, Sweden
joshua.entrop@ki.se

Lasse H. Jakobsen
Department of Hematology,
Clinical Cancer Research Center,
Aalborg University Hospital,
Aalborg, Denmark,
AND
Department of Mathematical Science,
Aalborg University,
Aalborg, Denmark
lasse.j@rn.dk

Michael J. Crowther
Red Door Analytics AB,
Stockholm, Sweden
michael@reddooranalytics.se

Mark Clements
Department of Medical Epidemiology and Biostatistics,
Karolinska Institutet,
Stockholm, Sweden
mark.clements@ki.se

Sandra Eloranta
Clinical Epidemiology Division,
Department of Medicine, Solna,
Karolinska Institutet,
Stockholm, Sweden
sandra.eloranta@ki.se

Caroline E. Dietrich
Clinical Epidemiology Division,
Department of Medicine, Solna,
Karolinska Institutet,
Stockholm, Sweden
AND
Red Door Analytics AB,
Stockholm, Sweden
caroline.dietrich@ki.se

*Corresponding Author

Appendix

A.1 Benchmarking

We calculated benchmark estimates of the mean number of events in scenario 1 to 9 as the expectation for the mean number of event function.

$$\mu(t|\boldsymbol{x}) = \int_0^t S(u|\boldsymbol{x})\lambda(u|\boldsymbol{x})du \tag{1}$$

Since we defined intensity and survival function to follow a Weibull distribution with known parameters defined in Section 3, we could re-write their expectations as follows for scenario 1 to 9:

$$\lambda(t|x_1) = \phi\rho\exp(0.4x_1)t^{\phi-1} \tag{2}$$

$$S(t|x_1) = \exp\{-[\iota\exp(1.2x_1)]t^{\psi}\} \tag{3}$$

Adding the continuous variable x_2 yields the following intensity and survival function used as the benchmark in the supplementary analysis presented in supplementary Table S1:

$$\lambda(t|x_1, x_2) = \phi\rho\exp(0.4x_1 + 0.8x_2)t^{\phi-1} \tag{4}$$

$$S(t|x_1, x_2) = \exp\{-[\iota\exp(1.2x_1 + 1.1x_2)]t^{\psi}\} \tag{5}$$

We used Rombergs method to approximate the integral of the product of the survival and intensity function as the same method was used in the implementation of our parametric estimator.

The benchmark estimates of the mean number of events in scenario 10 were obtained as the calculated mean number of events in the dataset of 10^6 observations, which was simulated based on the predefined parameters described in Section 3. An analytical approach for estimating the expectation of the mean number of events was not possible in this scenario as the intensity function of the recurrent event process depends on the number of previous events.

A.2 Figures

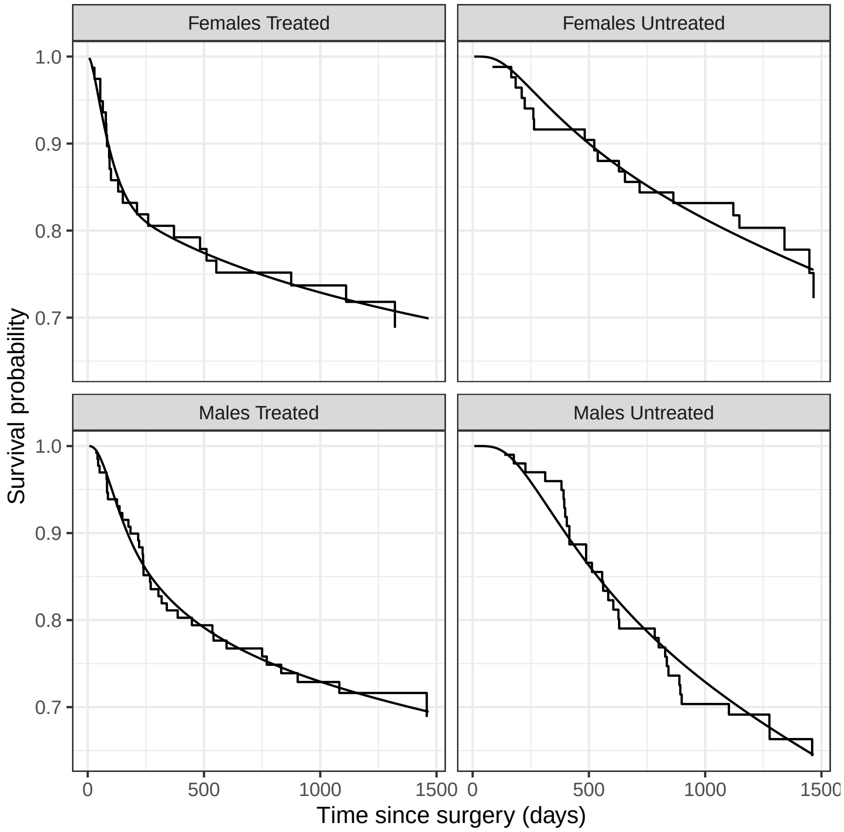

Figure S1: Survival after surgery for colon cancer by sex and chemotherapy treatment. The smooth curve represents estimation using flexible parametric models and the step function the non-parametric estimator.

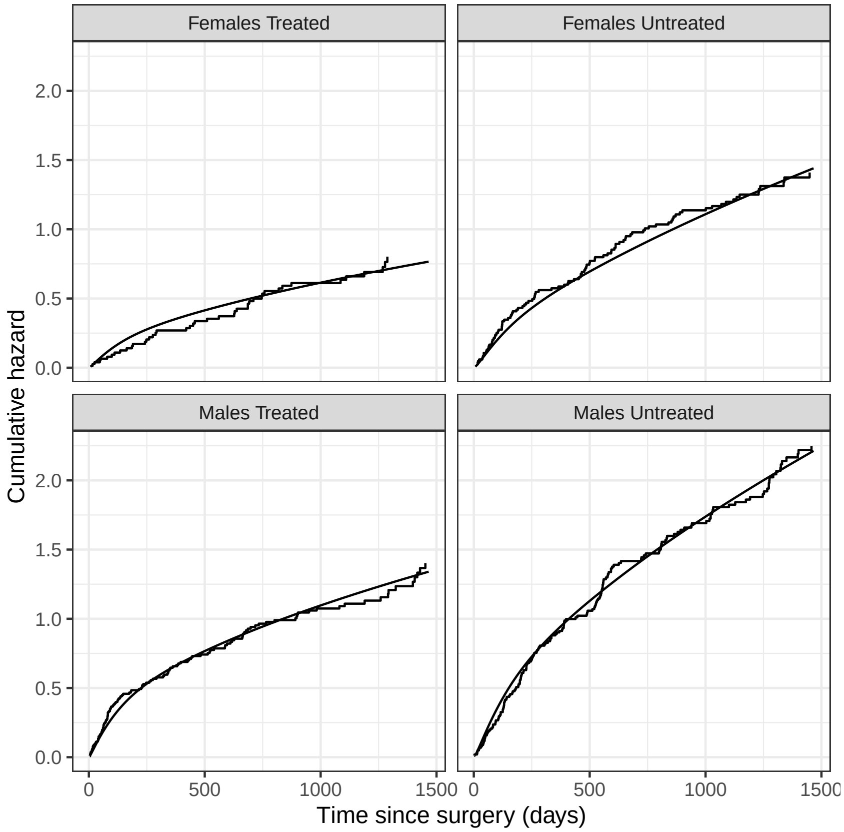

Figure S2: Cumulative hazard of rehospitalisations after surgery for colon cancer by sex and chemotherapy treatment. The smooth curve represents estimation using flexible parametric models and the step function the non-parametric estimator.

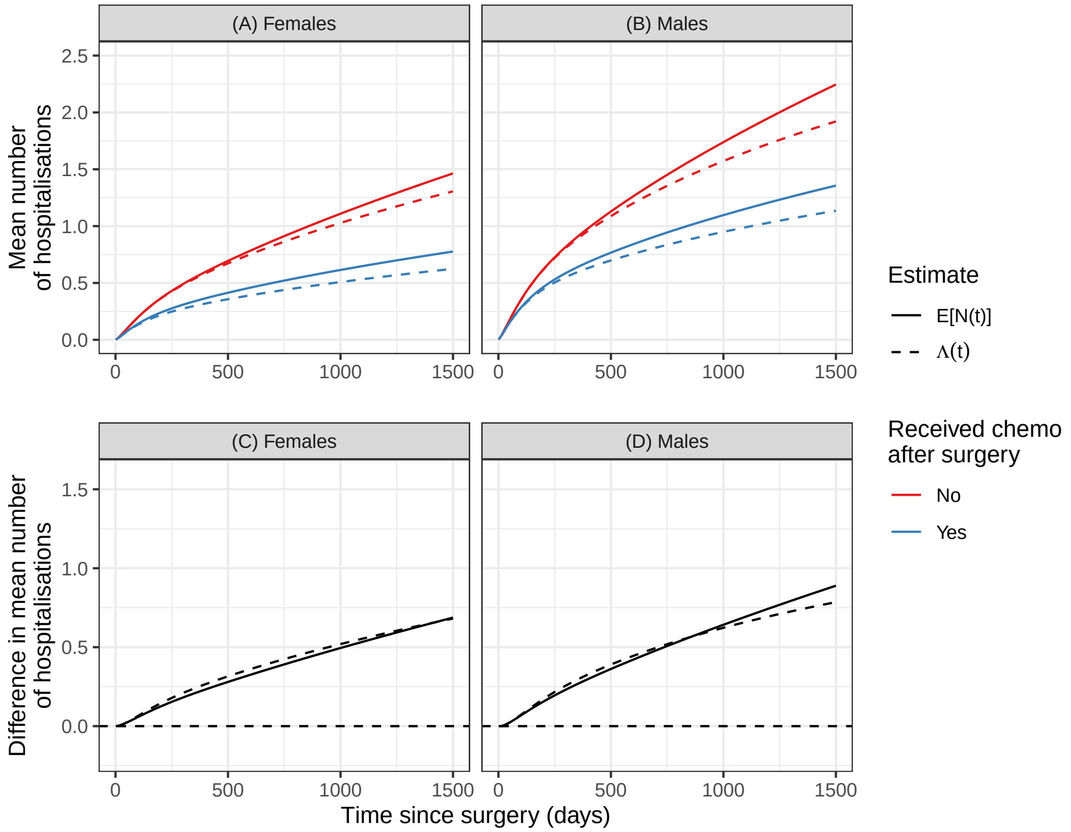

Figure S3: Net and cause-specific estimates of the mean number of rehospitalisations after surgery for colon cancer by sex and chemotherapy treatment. Solid lines represent cause-specific estimates of the mean number of rehospitalisations ($E[N(t)]$). Dashed lines represent net estimates of the mean number of rehospitalisations ($\Lambda(t)$), i.e., ignoring the competing risk of death.

A.3 Tables

Table S1: Estimates of bias, relative bias, and coverage at 2.5, 5, and 10 years of $\mu(t)$ in scenario 5 with an additional continuous variable x_2, which follows a standard normal distribution. Estimates highlighted in bold font were statistically significant different from their expected value at the 5% threshold based on their Monte-Carlo standard errors. Expected valued for bias, relative bias, and coverage were 0, 0%, and 95%, respectively.

		At 2.5 Years			At 5 Years			At 10 Years		
x_1	x_2	Bias	Rel. Bias	Coverage	Bias	Rel. Bias	Coverage	Bias	Rel. Bias	Coverage
0	0	-0.002	-0.181%	94.6%	-0.003	-0.163%	94.4%	-0.004	-0.088%	94.3%
1	0	0.002	0.078%	95.4%	0.005	0.090%	95.1%	0.024	0.189%	**93.9%**
0	1	**-0.005**	**-0.264%**	94.8%	-0.011	-0.266%	94.6%	-0.011	-0.126%	**93.7%**
1	1	0.003	0.068%	94.8%	0.019	0.187%	94.3%	**0.108**	**0.610%**	93.9%
0	2	-0.003	-0.103%	94.5%	0.017	0.300%	94.4%	**0.125**	**1.471%**	**93.4%**
1	2	0.045	0.691%	94.4%	**0.165**	**1.697%**	93.9%	**0.460**	**3.735%**	**93.5%**
0	3	**0.089**	**2.975%**	**93.6%**	**0.207**	**5.541%**	**92.8%**	**0.371**	**9.079%**	**92.5%**
1	3	**0.259**	**6.282%**	**92.6%**	**0.396**	**8.873%**	**92.3%**	**0.489**	**10.737%**	**92.2%**

Table S2: Estimates of bias, relative bias, and coverage at 2.5, 5, and 10 years of $\mu(t|x = 0) - \mu(t|x = 1)$. Estimates highlighted in bold font were statistically significant different from their expected value at the 5% threshold based on their Monte-Carlo standard errors. Expected valued for bias, relative bias, and coverage were 0, 0%, and 95%, respectively.

	At 2.5 Years			At 5 Years			At 10 Years		
Scenario	Bias	Rel. Bias	Coverage	Bias	Rel. Bias	Coverage	Bias	Rel. Bias	Coverage
**1**	**-0.004**	**0.453%**	94.9%	**-0.006**	**0.284%**	94.8%	-0.007	0.124%	94.8%
**2**	-0.001	0.023%	**93.7%**	-0.002	0.028%	94.2%	-0.013	0.079%	**93.6%**
**3**	**-0.008**	**0.211%**	94.9%	-0.011	0.106%	94.4%	0.002	-0.007%	95.0%
**4**	**0.003**	**-0.555%**	94.7%	**0.006**	**-0.488%**	94.6%	0.003	-0.100%	94.2%
**5**	-0.002	0.094%	94.6%	-0.006	0.149%	94.8%	-0.008	0.096%	94.9%
**6**	-0.003	0.116%	94.9%	**-0.017**	**0.266%**	94.4%	**-0.059**	**0.447%**	94.3%
**7**	**0.002**	**-0.747%**	94.3%	0.002	-0.422%	94.4%	0.000	-0.034%	94.5%
**8**	-0.001	0.120%	94.6%	0.002	-0.131%	94.7%	0.010	-0.581%	94.1%
**9**	**0.007**	**-0.524%**	94.4%	0.010	-0.479%	94.4%	0.014	-0.500%	94.1%
**10**	**-0.014**	**1.885%**	95.2%	**0.027**	**-1.348%**	94.5%	**-0.102**	**2.095%**	94.9%

Table S3: Estimates of bias, at 2.5, 5, and 10 years of Cook and Lawless' non-parametric estimator of the mean number of events with accompanying confidence intervals introduced by Ghosh and Lin. Estimates highlighted in bold font were statistically significant different from their expected value at the 5% threshold based on their Monte-Carlo standard errors. Expected valued for bias, relative bias, and coverage were 0, 0%, and 95%, respectively.

		At 2.5 Years			At 5 Years			At 10 Years		
Scenario	X	Bias	Rel. Bias	Coverage	Bias	Rel. Bias	Coverage	Bias	Rel. Bias	Coverage
**1**	0	0.000	0.030%	94.2%	-0.001	-0.159%	94.7%	-0.007	-0.307%	**91.9%**
	1	**0.004**	**0.303%**	94.0%	0.002	0.068%	94.6%	**-0.038**	**-0.490%**	**90.0%**
**2**	0	**-0.004**	**-0.348%**	95.6%	0.002	0.065%	94.6%	-0.013	-0.183%	**88.9%**
	1	-0.004	-0.114%	**96.3%**	**-0.014**	**-0.157%**	95.4%	**-0.079**	**-0.339%**	**91.1%**
**3**	0	0.001	0.034%	94.5%	0.004	0.092%	95.5%	-0.015	-0.126%	**89.4%**
	1	0.004	0.074%	95.2%	-0.002	-0.012%	94.9%	**-0.123**	**-0.316%**	**91.0%**
**4**	0	**0.002**	**0.616%**	94.5%	-0.002	-0.237%	95.1%	**-0.012**	**-0.729%**	**91.8%**
	1	**-0.002**	**-0.288%**	94.9%	**-0.006**	**-0.333%**	95.1%	**-0.035**	**-0.829%**	**91.2%**
**5**	0	0.002	0.229%	94.4%	**0.008**	**0.381%**	94.8%	-0.012	-0.250%	**91.2%**
	1	-0.001	-0.057%	94.0%	0.003	0.057%	94.7%	**-0.058**	**-0.457%**	**92.9%**
**6**	0	-0.002	-0.171%	94.5%	**-0.006**	**-0.177%**	95.5%	**-0.040**	**-0.501%**	**91.4%**
	1	0.001	0.017%	94.6%	-0.011	-0.113%	95.4%	**-0.154**	**-0.726%**	94.0%
**7**	0	0.001	0.331%	93.9%	-0.001	-0.237%	95.2%	**-0.008**	**-1.123%**	**91.2%**
	1	**-0.002**	**-0.446%**	**95.9%**	-0.002	-0.229%	95.2%	**-0.022**	**-1.746%**	**92.0%**
**8**	0	0.001	0.266%	95.4%	0.001	0.129%	95.4%	**-0.023**	**-1.131%**	**92.6%**
	1	0.000	-0.012%	95.5%	-0.006	-0.264%	94.9%	**-0.071**	**-1.907%**	**91.9%**
**9**	0	-0.002	-0.268%	94.6%	-0.003	-0.186%	94.6%	**-0.034**	**-0.997%**	**92.8%**
	1	**-0.010**	**-0.476%**	95.0%	**-0.020**	**-0.511%**	94.8%	**-0.137**	**-2.203%**	**92.1%**
**10**	0	0.000	-0.158%	95.1%	0.000	0.036%	94.9%	-0.007	-0.308%	**90.7%**
	1	0.000	-0.014%	94.8%	0.005	0.166%	94.8%	**-0.022**	**-0.313%**	**93.6%**

Table S4: Estimates of bias at 2.5, 5, and 10 years when estimating the mean number of events ignoring the competing event.

		At 2.5 Years		At 5 Years		At 10 Years	
Scenario	X	Bias	Rel. Bias	Bias	Rel. Bias	Bias	Rel. Bias
**1**	0	0.009	2.593%	0.032	3.501%	0.116	4.841%
	1	0.043	3.743%	0.155	5.155%	0.562	7.222%
**2**	0	0.023	2.199%	0.090	3.261%	0.346	4.811%
	1	0.118	3.412%	0.450	4.981%	1.697	7.269%
**3**	0	0.043	2.460%	0.156	3.385%	0.566	4.725%
	1	0.213	3.675%	0.768	5.106%	2.793	7.179%
**4**	0	0.075	26.111%	0.264	38.328%	0.921	57.941%
	1	0.348	40.998%	1.205	61.757%	4.092	96.470%
**5**	0	0.224	26.084%	0.795	38.557%	2.769	58.056%
	1	1.045	40.969%	3.630	62.018%	12.291	96.593%
**6**	0	0.371	25.924%	1.322	38.452%	4.619	58.109%
	1	1.736	40.854%	6.046	61.970%	20.517	96.749%
**7**	0	0.178	97.282%	0.583	158.374%	1.839	273.388%
	1	0.754	170.973%	2.363	298.733%	7.079	567.455%
**8**	0	0.532	97.017%	1.744	157.793%	5.498	272.517%
	1	2.269	171.559%	7.100	299.178%	21.255	567.974%
**9**	0	0.890	97.347%	2.922	158.662%	9.224	274.318%
	1	3.770	171.003%	11.828	299.031%	35.468	568.656%
**10**	0	0.067	21.584%	0.331	40.252%	1.361	61.558%
	1	0.474	45.201%	1.832	64.518%	7.382	104.050%
